# Patient participation in palliative care decisions: An ethnographic discourse analysis

**DOI:** 10.3402/qhw.v11.32438

**Published:** 2016-11-22

**Authors:** Emmanuelle Bélanger, Charo Rodríguez, Danielle Groleau, France Légaré, Mary Ellen MacDonald, Robert Marchand

**Affiliations:** 1 Department of Social and Preventive Medicine, Public Health Research Institute (IRSPUM), Université de Montréal, Montréal, Canada; 2 Department of Family Medicine, McGill University, Montreal, Canada; 3 Department of Psychiatry, McGill University, Montreal, Canada; 4 Department of Emergency and Family Medicine, Université Laval, Quebec City, Canada; 5 Division of Oral Health and Society, McGill University, Montreal, Canada; 6 Department of Family and Emergency Medicine, Université de Montréal, Montreal, Canada

**Keywords:** Palliative care, decision-making, patient participation, qualitative research, discourse analysis

## Abstract

The participation of patients in making decisions about their care is especially important towards the end of life because palliative care decisions involve extensive uncertainty and are heavily influenced by personal values. Yet, there is a scarcity of studies directly observing clinical interactions between palliative patients and their health care providers. In this study, we aimed to understand how patient participation in palliative care decisions is constructed through discourse in a community hospital-based palliative care team. This qualitative study combined ethnographic observations of a palliative care team with discourse analysis. Eighteen palliative care patients with cancer diagnoses, six family physicians, and two nurses were involved in the study. Multiple interactions were observed between each patient and health care providers over the course of 1 year, for a total of 101 consultations, 24 of which were audio-recorded. The analysis consisted in looking for the interpretive repertoires (i.e., familiar lines of argument used to justify actions) that were used to justify patient participation in decision-making during clinical interactions, as well as exploring their implications for decision roles and end-of-life care. Patients and their health care providers seldom addressed their decision-making roles explicitly. Rather, they constructed patient participation in palliative care decisions in a covert manner. Four interpretive repertoires were used to justify patient participation: (1) exposing uncertainty, (2) co-constructing patient preferences, (3) affirming patient autonomy, and finally (4) upholding the authority of health care providers. The results demonstrate how patients and health care providers used these arguments to negotiate their respective roles in decision-making. In conclusion, patients and health care providers used a variety of interpretive repertoires to covertly negotiate their roles in decision-making, and to legitimize decisions that shaped patients’ dying trajectories. Discourse analysis encourages awareness of the role of language in either promoting or hindering patient participation in decision-making.

Patient involvement in decision-making regarding their care has been advocated as a cornerstone of person-centred medicine (Barry & Edgman-Levitan, 2012; Munthe, Sandman & Cutas, 2012). Patient participation in decision-making is deemed particularly appropriate towards the end of life because these decisions are preference-sensitive, involve significant uncertainty, and often present medically equivalent treatment options, a notion known as equipoise (Elwyn, Frosch, & Rollnick, 2009; Müller-Engelmann et al., 2013). Equipoise refers to decisions that cannot be made with the evidence base alone and that require the elucidation and integration of patient preferences, such as the decision to undergo palliative chemotherapy or to pursue prophylactic anticoagulation in the event of advanced cancer. Palliative care consists of a holistic and interdisciplinary approach to care that seeks to improve the quality of life of patients and their families when confronted with a life-threatening illness. The bulk of research on patient participation in palliative care decisions has focused on decision role preferences (Bélanger, Rodríguez, & Groleau, 2011; Gaston & Mitchell, 2005). This literature suggests that a majority of palliative care patients prefer a shared or active role in decision-making. However, there is a paucity of research directly observing decision-making interactions in palliative care (Bélanger et al., 2011; Fine, Reid, Shengelia, & Adelman, 2010). Existing observational studies have been limited to checking the content of consultations for the presence of explicit information and encouragement for patient participation and to providing concomitant descriptive statistics (Gattellari, Voigt, & Butow, 2002; Timmermans et al., 2005; Timmermans, van der Maazen, Leer, & Kraaimaat, 2006; Verhaak, Kraaimaat, Staps, & van Daal, 2000).

The phenomenon of shared decision-making (SDM) has been conceptualized in different ways (Makoul & Clayman, 2006; Moumjid, Gafni, Brémond, & Carrère, 2007; Wollschläger, 2012). At a minimum, SDM has been defined as the general process of sharing information and negotiating treatment options with patients before reaching a decision (Charles, Gafni, & Whelan, 1997; Cribb & Entwistle, 2011). A great deal of research has focused on decision antecedents, that is, on eliciting and respecting patient preferences for either an active, shared, or passive decision role (Scholl et al., 2011). A prescriptive construct of SDM has also emerged from evidence-based medicine (EBM), with an emphasis on integrating both the strongest evidence with patients' values when making decisions (Légaré & Witteman, 2013). Even when SDM is approached as the bridge between EBM and patient preferences through the use of formal decision support tools, there remains concern about discourse, particularly how these tools could be presented to support clinicians' recommendations rather than elicit patient involvement. Patient participation in decision-making can therefore also be conceptualised as an interactive process, whereby power over decision-making is constructed and legitimised through discourse during social interactions (Robertson, Moir, Skelton, Dowell, & Cowan, 2011; McMullen, 2012). We adopted the latter conceptualisation to explore the aspects of discourse that construct patient participation and how palliative care physicians share power with patients through talk, with an emphasis on aspects of conversations that cannot be captured with checklists on the explicit communicative elements of SDM.

Although discourse analysis is a promising methodology for studying difficult conversations about decision-making towards the end of life (Bakitas, Kryworuchko, Matlock, & Volandes, 2011; O'Connor & Payne, 2006), the ways in which patient participation in palliative care decisions occurs through discourse have yet to be studied. Medical sociologists have made significant contributions to our understanding of the asymmetry of medical consultations in other clinical contexts, by demonstrating how these power differences are reproduced during talk in interaction (Maynard, 1991; Ten Have, 1991). More specifically, authors using this methodology focus on specific conversational sequences that physicians use during decision-making (Collins, Drew, Watt, & Entwistle, 2005; Peräkylä, Lindfors, & Ruusuvuori, 2007), such as offering a choice (Toerien, Shaw, Duncan, & Reuber, 2011), responding to medical recommendations (Koenig, 2011), and displaying compliance and expertise (Barton, 2000). However, studies in the tradition of conversation analysis focus on the sequential organization of very specific aspects of talk. They thus cannot offer an overall assessment of patient participation in decision-making beyond specific conversation sequences, and they do not engage with existing conceptual models of decision-making (Peräkylä et al., 2007). Consequently, there remain a number of research gaps on patient participation in palliative care decisions, as well as a lack of observational studies and longitudinal designs examining recurring decisions in the context of lasting therapeutic relationships. To fulfil this knowledge gap, we stated the question addressed in this investigation as follows: In the context of a hospital-based palliative care team, how do health care providers and their patients discursively construct patient participation in palliative care decisions? This research offers a description and interpretation of the discourse used to construct patient participation in decision-making, namely the types of discursive practices used to share power with patients during decision-making interactions.

## Methodological approach

In this article, we adopt a social constructionist epistemological position (Gergen, 1985), positing that language does not merely reflect social reality, but actively constructs it. Drawing on critical discursive psychology (Wetherell, 1998) and Foucault's work as theoretical background (Foucault, 1971, 2007; Veyne, 2008), we further uphold this assumption by emphasizing the importance of discourse in reproducing power relations. We conceptualize patient participation as the discursive process of sharing power over decision-making during social interactions. The research methodologies selected for this study were organizational ethnography (Neyland, 2008) and discourse analysis as developed by social psychologists (Potter, 2011; Potter & Wetherell, 1987). Ethnography is an iterative research process that involves sustained contact with a group of participants in the context of their daily lives (Green & Thorogood, 2009). Participant observation allowed in-depth exposure to the sensitive discourse of patients and health care providers making palliative care decisions in their organizational situated context. Discursive psychology emphasizes the contextual and relational nature of attitudes and identities (Jørgensen & Phillips, 2002). Unlike cognitive approaches to psychology, discursive psychology addresses the inconsistencies that appear when people create different versions of events through talk, as well as their social consequences (Potter, 2011; Potter & Wetherell, 1987). Discursive psychology is particularly suited to analyse the structure of discourses that construct patient participation in decision-making as acceptable, as well as the types of arguments that legitimize it in clinical practice. This methodological combination is particularly suitable to analyse the power dynamics involved in the performance of decision-making roles during clinical interactions in a given organizational context.

### Methods of Data Generation and Study Participants

Data were generated over one year of participant observation in the palliative care service of a community acute-care facility located in Montreal, Canada, from April 2010 to April 2011. Patient–physician dyads were the primary unit of analysis, and patients were followed longitudinally through the course of their care and decision-making processes. Patients who were deemed cognitively able by their palliative care physicians and who spoke French or English were eligible to participate in this study. We recruited patients under the care of all health care providers in the team. A total of 30 patients verbally agreed to participate in the study; however, seven deteriorated too rapidly or were discharged to another facility before signing the consent form. Five additional patients signed consent forms but were not retained in the analysis because of insufficient data after being discharged or experiencing a rapid decline in their ability to participate in decisions. Over a period of one year, 18 patients were followed through the course of their care across different settings. Of the 18 patients retained, 15 were deceased by the end of the field research. The patients all had advanced cancer, and approximately one-third also had serious co-morbidities, such as heart failure and chronic pulmonary obstructive disease. Ten patients were women; eight were men. Their ages ranged from 54 to 88, with an average age of 70. The consultations addressed a variety of decisions such as anticoagulation, pain control and place of care, many of which were revisited over time. An average of seven consultations were observed per patient. The range was between 1 and 14, and this was directly related to patients' lifespan.

The palliative care service under study was mobile across a community hospital, and also followed palliative care patients in the outpatient clinic and at home (Bélanger, Rodríguez, Groleau et al., 2014). Health care providers included two pivot nurses who were responsible for overseeing the continuum of care and six family physicians who were each responsible for hospitalized patients for one week and who followed their own patients at home and at the outpatient clinic unless an exacerbation required hospitalization. There were no specialized palliative care nurses providing inpatient care at the hospital. Most referrals came from the oncology department, although emergency services, the intensive care unit and family physicians in the community also referred patients. Administrative data indicated that 192 patients died at the hospital while being under the care of the team in 2009 and that 53 died at home, and 30 were transferred to neighbouring stand-alone palliative care units.

The field researcher (first author) was present at the hospital every weekday from 9:00 am to 5:00 pm at a minimum. The aim of the participant observation was to have access to naturally occurring decision-making processes as they occurred over time, paying particular attention to how the organization of care influenced decision-making processes, and how decisions were initiated and framed during and outside of consultations. Observations included both formal and informal inpatient, outpatient and home consultations during which participating patients and palliative care physicians made decisions. Observations also targeted the work of the palliative care team beyond consultations, including weekly interdisciplinary meetings, daily hospital rounds, lectures to residents and informal discussions between consultations. Formal consultations with participating patients were audio-recorded with permission whenever possible and appropriate. The field researcher kept detailed field notes of all observations made during consultations, meetings and after informal conversations. In total, 101 consultations were observed between patients and their health care providers, 24 of which were audio-recorded with the patients' permission. When the audio-recorder was not used, the field notes concerned detailed aspects of discourse (initiation of decision-making processes, sequence of arguments, sequence of utterances, etc.) all written short-hand and transcribed immediately after consultations. A reflexive journal was also kept throughout fieldwork to consider the researcher's influence over fieldwork and analysis.

The study was approved by the ethical review boards at McGill University Faculty of Medicine and the hospital where the study took place. The ethnographic research design was adapted to the sensitive clinical situations in several ways. Only short informal interviews were carried out with patients outside of consultations, in order to prevent depleting their energy. A clear and concise consent form was used with recruited patients during the first consultation to ensure that they understood the nature of their participation and their right to withdraw at all times without any consequence for the care provided. Continued consent was revisited over time, especially if patients' cognitive status fluctuated. The field researcher had extensive experience as a palliative care volunteer and was prepared for this delicate context of care. Consent forms also made very clear that information obtained about participants would remain anonymous.

### Methods of data analysis

The field researcher first carefully transcribed the audio recordings of all observed consultations. The material was then organized into decision exchanges: that is into interactions about any therapeutic actions, such as initiating, modifying, or stopping a medication or procedure. The consultations contained many decision exchanges. The field notes on decision-making interactions were also analyzed to contextualize the decision-making processes in the organizational culture of the palliative care team. The ethnographic observations were instrumental to our description of the organizational context and our interpretation of discursive practices, especially when health care providers volunteered their interpretations of the interactions in informal discussions. In terms of discursive practices, we searched for “interpretive repertoires,” a term coined by Potter and Wetherell (1987), and defined as familiar and habitual lines of argument or terms drawn on to characterize or evaluate action. In this study, interpretive repertoires were ways of talking used by clinicians and patients to construct patient participation in decision-making and present it as acceptable. Discursive psychology also considers the social consequences of discourse for the positioning of speakers involved in a given interaction. We thus explored the ways in which interpretive repertoires justified the decision roles of palliative care patients and their health care providers, and how these decisions affected the way in which palliative care patients ultimately died. The first author coded the data with the help of the HyperRESEARCH 3 qualitative data analysis software, and the interpretation then occurred over several iterations following feedback from the co-authors. Data were analyzed in the original language (See Appendix A for transcription details) and excerpts were translated into English as needed; these excerpts provide examples of recurring patterns throughout the data.

## Results

### Interpretive repertoires

Sustained observation of the interactions between patients and health care providers indicated that both groups seldom discussed their decision roles overtly. In other words, palliative care providers did not explicitly ask patients whether they wanted to participate in decision-making, and patients did not spontaneously express preferences for decision roles. Rather, both patients and health care providers drew on a set of four interpretive repertoires to justify patient participation, thus making specific decisions and their respective decision roles acceptable. Table 1 presents the list of repertoires with several examples of their use by health care providers and patients. The repertoires were closely related to the organizational culture of the palliative care team. In the results below, we present the four repertoires in turn and rely on ethnographic field notes to situate and interpret the discursive practices in their organizational context.

**Table 1:** Examples of Interpretive Repertoires Used by Health Care Providers and Patients

Repertoire	Health care providers	Patients
Expose Uncertainty	“I don't have the answer.” “The treatment could help, but it could also make you sicker” “We will never know what the best decision is.” “Some patients prefer X, but some prefer Y” “Your physicians disagree.”	“The doctors don't even know how much time I have left.” “If we don't know that it won't make me sicker then I'd prefer …”
Co-construct patient	“Do you feel up to telling me what you think?”	“Do I have to go to the hospital for this?”
preferences	“You've had this treatment before, how do you feel about continuing?”	“What happens if he bleeds out?” “This happened in the past.”
	“Do these symptoms bother you?”	“You're not going to do this to me again.”
	“What makes you say that you would like to stop this treatment”	“Is there something that can be done about this symptom?”
	“Would you like to go home? Has this been discussed?”	“I've made some adjustments in my medication that I'd like to talk about.”
	“This treatment means X, what do you think about that?”	“Last time I had no side effects.” “When my relative had this treatment …”
	“You have a choice, which one do you prefer?”	
Affirming patient	“It's your life, your decision”	“You may not believe me, but I'm in pain.”
autonomy	“What matters is to do what you want.”	“If I were obliged to have this treatment ….”
	“You always have a choice.”	“I never have a choice, I just do what I'm told.”
	“We are here so that you feel supported in your decision.”	“I feel trapped.”
	“You should do what you want.”	
	“You can do whatever you want with your medication.”	
Upholding the	“My medical expertise tells me that …”	“I'd rather my physician decides”
authority of health care providers	“I will make the decision when it's time to go to the hospital.”	“You're the doctor.” “What would you do?”
	“I do not recommend this treatment for someone with cancer.”	“I wanted your blessing” “You have to make a decision.”

#### Repertoire #1: exposing uncertainty

Palliative care providers recognized that they were practicing in a clinical context fraught with uncertainty. During interdisciplinary meetings, they mentioned that clinical evidence with their specific patient population was lacking in many important areas of decision-making. Patients and their family members frequently cared about outcomes that were difficult to quantify, that had not been researched, or that could only be achieved through undesirable trade-offs. For example, certain types of palliative chemotherapy might lengthen life expectancy while reducing quality of life because of side effects. Although the timing and specific cause of death remained unpredictable, the deterioration and death of patients was ultimately a certainty. This context shaped how uncertainty was exposed in the discourse of palliative care providers, who were committed to accompanying patients and their families through their uncertain future.

As a repertoire, exposing uncertainty encompassed either admitting an inability to designate a superior option on the basis of medical evidence or presenting options as equally justifiable in practice. Uncertainty represented a justification for the patients’ involvement in decision-making. Patients mentioned uncertainty to justify their preferences, for example, by referring to the uncertainty of their life expectancy when justifying the refusal of palliative chemotherapy: “So basically they don't even know how much time I have left.” The repertoire undermined the medical rationale for choosing one option over another, making it acceptable for the patient to express an opinion during the decision-making process.

**Excerpt 1: Decision about anticoagulation, home consultation based on audio recording**

Patient (P): I went to eh for, my pace maker.

Doctor (Dr): Yeah.

P: And then eh, the doctor the first thing he asked me, he says, when did you stop your warfarin? […]

Dr: (checking in papers) We are swimming a little in eh unknown waters. At some point we found that it was more dangerous to take it than not to take it. […] It was because of your atrial fibrillation, the atrial fibrillation, there is about a 5% risk having a cerebrovascular accident per year (pause) eh.

P: That's why we stopped the warfarin?

Dr: We stopped it because at some point you go much worse, you were not in well and eh in the context of cancer we know that warfarin is not great, it doesn't work great, that, that's why for some time you had injections. Do you remember?

P: Yeah.

Dr: That are much better than warfarin, so you took heparin (tinzaparin) at 16,000 units and then we discussed this together to know what are the risks and benefits of taking it in a man who had eh, with cancer, hm, who was mobile, who moves well, whose risk of phlebitis and thrombophlebitis were not too big, and for whom the risk of, of, of, of stroke, of emboli of cardiac origin were around 5 or 6% so we had decided to stop […] (reading) anticoagulated with warfarin. Does he really need it? In my opinion the risks were outweighing the benefits at that point (pause) because I find that the risks of complications with warfarin are high and the risks of prevention, it does, it doesn't really prevent. Eh the thrombophlebitis, on the other side if you want to take the heparin like before.

P: What will that do?

Dr: It prevents it protects you it reduces the risks of having stroke, of having a cerebrovascular accident.

P: Ok.

Dr: And paralysis (pause) […]

P: I can't give you the answers.

Dr: It was me who stopped it. (pause) The reason is simple I don't have the answer.

P: I asked questions to the nurse and she didn't know either.

Dr: The answer clearly she knows it less than I do. I know that there are advantages and disadvantages. The advantages are to reduce the risks of having a paralysis eh a small blood clot in your brain or a clot in your legs that goes to your lungs, to reduce this you have be ready to take.

P: These injections.

Dr: To me the only thing I will give you and that is worth taking is eh, the heparin.

P: But that they had started it in the hospital?

Dr: hm-hm, hm-hm, we stopped it because we told ourselves well, it may be a little advantageous but there are risks to bleed out. Have you ever spit up blood?

P: Never.

Dr: Never.

P: Never blood in my stool.

Dr: hm […] I don't have the answer for the injection. I don't have the answer.

P: If it's to protect.

Dr: Now are you going to go get, are you getting more chemo?

P: No.

Dr: That's it cancer of the colon with peritoneal carcinomatosis.

P: I stopped when I went to the hospital it was over then.

Dr: I think if you are a bit less mobile I will restart the anticoagulants.

P: The what?

Dr: If you move a little bit less at some point you are weaker.

P: Ah no, now I'm in shape.

Dr: I know.

P: I'm in really good shape [description of daily activities omitted]

Dr: It's difficult. I don't have the one good answer. You are doing well? We continue like this?

P: I think so. I rest. We both rest. I think we have a good system.

Excerpt 1 above offers an example of the uncertainty repertoire used during a decision-making interaction regarding the use of anticoagulants to reduce the risk of stroke in a patient with atrial fibrillation. The physician repeatedly asserted that he did not have the answer to the question of whether using anticoagulants was the best option for the patient. The discussion involved considerable back and forth, and the statements about uncertainty clearly conveyed that patient participation was expected, as evidenced by the patients’ response: “I can't give you the answers”. The decision was ultimately resolved by exploring the patients’ daily activities. It was common for health care providers to acknowledge the existence of uncertainty during decision-making conversations. For example, during an inpatient consultation about the use of antibiotics to treat a recurring pneumonia, a physician said: “Some people in those circumstances will say it's easy, we treat and that's it, but it isn't as simple as always treating because sometimes it doesn't work.” The uncertainty of the outcome of treatment therefore justified patient involvement in the decision-making process. The options were then presented as equal by providing examples of patients who selected either option: “It's a decision that is hard for me to make alone because some people will say yes every time, we treat them and we buy another 2 weeks, while others say that they have had enough with being sick and only want to be comfortable to leave.” Assertions such as: “I cannot say alone what the best is for you” constructed the participation of the patient as crucial and led to direct prompts for the patient's opinions. It is important to note that, when uncertainty had been acknowledged, a lack of patient contribution was regarded as problematic by the treating team. When this happened, the ethnographic observations reveal that the health care providers volunteered justifications to the field researcher, saying, for example, that some patients were afraid of dying. In effect, the repertoire of exposing uncertainty called for patients to express a preference, meaning that a lack of patient participation made it difficult for health care professionals to justify choosing one option over another. Overall, the interpretive repertoire of uncertainty encouraged patient participation by acknowledging the limits of medical knowledge regarding treatment options.

#### Repertoire # 2: co-constructing patient preferences

From the way palliative care providers taught students and the types of concerns that were discussed during interactions among team members, it was apparent that maintaining quality of life, as defined by the patient, constituted a paramount goal of care. This focus on comfort and pain management was part of the philosophy and culture of palliative care, setting it apart from other medical specialties and from the aggressive treatments that patients endured during the curative phase of their illness. Respecting patients’ ideas about quality of life therefore represented a way of reorienting care when interventions became unlikely to affect the life expectancy of patients.

A very common interpretive repertoire, co-constructing patient preferences consisted in discussing different options and treatment modalities to create a preference and legitimize a decision. Most patients did not come to the consultations with pre-existing preferences for treatment options. In fact, their reactions suggested that they did not even anticipate the decisions that they would face in palliative care. In this repertoire, professional recommendations were framed as suggestions or questions, and accompanied with direct prompts for patients’ opinions when discussing treatment modalities.

**Excerpt 2: Decision about neurological investigation, inpatient consultation based on field notes**

Doctor (Dr): The nurse called me too to tell me that you were like gone.

Patient (P): Me?

Dr: No not you, you don't have my phone number at home (laugh).

P: That's what I thought.

Dr: So the nurses were worried because you weren't responding but you had your eyes open. Has this ever happened at home?

P: Yes. Sometimes at home I'm gone like that, when I watch TV and I don't watch anything unsettling and then all of a sudden I'm not there anymore.

Dr: Has this being going on for a long time?

P: A year maybe.

Dr: Have you talked about it with your family doctor?

P: I don't know.

Dr: Ok, are you tired after?

P: No a bit confused, dizzy.

Dr: Is this something that bothers you a lot these absences?

P: No it's part of my life. My partner worries about it a bit but not me. It doesn't annoy me enough to want to know.

Dr: It's like a free trip (laugh). It's because it could be epilepsy, so that's what worries me the most.

P: That's when people fall down?

Dr: No there are 100 different ways of having an epilepsy crisis, some people fall down and lose consciousness but it can also manifest itself as an absence like you. If I am curious and I would like to have you seen by a neurologist, would you accept?

P: If she can come while I am here yes, but I'm not going to stay another week for that.

Dr: So it's not something that you would like us to examine in more depth to try to treat it?

P: No, I don't want to stay any longer. It's long here in the hospital.

Dr: I understand and you come to see us often. Ok, so I will see if there is something that can be done while you are here and that we make sure that you are stable but making sure that we are not making your hospital stay any longer, because anyway as you know it's not worth leaving through one door and coming back through the next.

P: No.

In the data analyzed, patients rarely volunteered the expression of preferences without providing a justification along with their preference. In the excerpt above, the patient's refusal to do more neurological tests is accompanied by an explanation that she had already been in the hospital too long, which is supported by her medical history. Patients’ past clinical experiences were among the most powerful arguments used to justify a preference. When it came to constructing preferences for specific options, narratives about the end of life of relatives and references to physical symptoms experienced during previous interventions also constituted acceptable justifications. Patients largely relied on these types of arguments rather than on medical facts and evidence to justify their preferences for specific options. These assertions were never challenged as such by the health care providers; rather, new interventions had to be constructed as different from previous ones to undermine the applicability of previous experiences. The palliative care options discussed were only acceptable when supported by patients. Rhetorically, interventions that were not supported by patients became difficult to defend. In summary, the repertoire of co-constructing patient preferences for different options encompassed an accessible resource for physicians who wished to negotiate and validate treatment plans, as well as for patients who thus contributed to or undermined the legitimacy of a given option.

#### Repertoire #3: affirming patient autonomy

In the current practice of palliative care, dying tends to be approached as an individual experience rather than as a social or religious one as it was in the past (Kellehear, 2007; McNamara, 2004). Involving patients in deciding how they would like their death to unfold is thus very important in helping patients make sense of their end of life. During the observation of interdisciplinary meetings, there appeared to be an absence of consensus about what it meant to have a good, meaningful death, so professionals deferred to patients on this matter. This context explains the importance of affirming patient autonomy when making palliative care decisions.

The third repertoire concerned the affirmation of patient autonomy, and it was often promoted for its own sake or justified with legal vocabulary. Utterances such as “what matters is to respect your wishes” appeared as self-justifying accounts of patient autonomy. These accounts represented a normative depiction of events under the control of the patient. Legal vocabulary was also used to secure the voice of the patient; for example, one physician reminded a patient that she had the “right” to say no to various interventions. In addition, health care providers would sometimes reassure patients that whatever decision they made would be the right one. Conversely, from the perspective of patients, claiming a powerless position implied a lack of respect for patient autonomy and triggered a shift back to the second repertoire of co-constructing patient preferences. The following excerpt offers an example of this repertoire.

**Excerpt 3: Decision about urinary catheter, inpatient consultation based on field notes**

Doctor (Dr): I think that we'll have to put in a catheter again.

Patient (P): I never have a choice about anything; I do what I'm told.

Dr: You always have a choice, but you cannot stay like this without being able to empty your bladder. It can damage your kidneys and you can have urinary tract infections Do you prefer to have a catheter (bag) or to do catheterisations?

P: A bag.

Dr: Well in the hospital we'll put in a catheter and then later when you go home we'll see what we can do.

In this excerpt, a resigned patient stated: “I never have a choice about anything. I do what I'm told”. These types of affirmations yielded strong reactions form health care providers who reasserted the importance of patient autonomy. In another instance, during a discussion about blood transfusions to manage anaemia, a patient suffering from uterine cancer and persistent vaginal bleeding stated “If I were obliged to do so,” and was quickly interrupted by the physician telling her that she was never obliged to do anything. Thus, patient autonomy was an argument that established the patient as a legitimate source of authority during decision-making.

#### Repertoire #4: upholding the authority of health care providers

Palliative care providers rely on a number of legitimate sources of authority, mainly regarding their medical expertise. Palliative medicine is a context of care where patients are particularly vulnerable. As a result, participating physicians were wary of imposing participation onto patients and communicated this explicitly when teaching residents, warning them against pressuring patients into making decisions on their own.

The final repertoire present in the data related to upholding the authority of health care providers. Although this repertoire served as a strategy for patients to defer decision-making to physicians, it was also used by patients as a means of re-establishing respect for physician's authority in the rare event that patients expressed preferences without being prompted to do so. Excerpt 4 shows that a patient expressed a desire to keep his pain medication unchanged: “You're not going to lower them, are you?”, but ultimately ended with a reassertion of his trust in his physician: “It's alright, you are the doctor”. There were several utterances that served this purpose, usually emphasizing patients’ lack of medical knowledge or training compared to that of physicians.

**Excerpt 4: Decision about pain medication, outpatient consultation based on field notes**

Doctor (Dr): So every day, 4 hydromorphone and 2 oxycodone. I will let you finish this but we will have to make adjustments, because now you have two medications for pain. We'll have to take care of that.

Patient (P): You're not going to lower them, are you?

Dr: Where is your pain?

P: Everywhere, it's depression too. All that together, it gives me a hard time getting started in the morning.

Dr: Why don't you take them every 4 hours? Because it's a lot two. I will leave it that way, but next time we'll have to take care of it.

P: It's all right, you are the doctor.

Physicians also upheld their authority when making recommendations: “My medical expertise tells me that in people who have cancer like you, when there is a minor complication and we treat it, it works quite well.” However, health care providers mainly drew on traditional sources of authority when patients exhibited physical and verbal signs of distress. During the fieldwork, distress was most commonly observed during the days and hours leading up to the patients’ deaths. For instance, when a patient appeared distressed about the burden of home care for her family during her last days of life, the nurse replied: “If it's not going well, I will be the one deciding that it's time to go to the hospital.” To summarize, the repertoire of upholding the authority of health care providers represented a way for patients to defer decision-making responsibilities or to mitigate what could be perceived as a challenge to physicians’ authority, as well as a means for clinicians to resolve decision-making swiftly when patients exhibited distress.

### Implications for decision roles and the end of life

The four interpretive repertoires identified in this study constitute the discursive practices that were used by speakers to justify patient participation in decision-making and thus negotiate decision roles during the interaction. Although these were examined separately for analytical purposes, the construction of patient participation in decision-making involved many repertoires successively. For example, a physician would present the uncertainty of any given course of treatment, establish the treatment modalities of a particular treatment option as acceptable through a discussion of patient experiences or daily life, emphasize the intrinsic autonomy of patients, and then resist the patients’ attempts to defer decision responsibility back to clinicians. Control over decision-making interactions was often shared, but also at times contested or avoided. Power over decision-making was shared by exposing uncertainty and then co-constructing patient participation through direct prompts. Patients’ attempts at deferring decision-making to health care professionals were countered with all three other repertoires: reiterating the existence of uncertainty, attempting to construct a preference for treatment modalities, or affirming patient autonomy. In contrast, power over decision-making was contested in the absence of the repertoire about uncertainty; patients’ preferences then clashed with those of health care providers. When patients did not participate in decision-making, the field notes show that health care providers volunteered justifications to the field researcher, such as emphasising their concern for the risk of complication depending on the outcome of the decision.

Excerpt 5 below offers an example of both patient and health care provider avoiding control over decision-making. A patient with stomach cancer had suffered a heart attack, which was a rare complication of palliative chemotherapy, and a stent had been installed during emergency heart surgery. He was now faced with a decision about the use of anticoagulation medication, which could help prevent the heart stent from blocking, but could also increase the risk of bleeding from stomach cancer. This patient had a short life expectancy either way; so the decision concerned how he preferred to die.

**Excerpt 5: Decision about anticoagulation to prevent stent from blocking, home consultation based on field notes**

Doctor (Dr): As far as the anticoagulants are concerned, the problem is that your physicians disagree. It's obvious that I have the perspective of a palliative care physician, but on the other hand, now that we put in a stent we might as well keep it open. We could continue for a month and follow you closely if you do not bleed. We are here so that you feel supported in your decision.

Patient (P): I don't know […]

Dr: It is important that you understand the risks. One has to know that if you continue taking the anticoagulants you will need a blood test every week. If your haemoglobin decreases too much we stop taking them. Do you feel up to telling me what you think?

P: I want to put the chances on my side. I have been so sick. It makes me nervous.

Dr: The choice is yours to make; what do you think of a blood test every week?

P: It doesn't matter.

Family: If he bleeds, what does it mean?

Dr: We are not talking about a massive hemorrhage, but you would be weaker and you could need to go to the hospital for a blood transfusion, for that you need to go to the hospital. And it is possible that he bleeds to the point of endangering his life.

Family: So if it is a serious haemorrhage he could die from it?

P: I would like it better if my physician decided.

Dr: Yes but it is your life, your decision. You can wait a little also before deciding, take a few days and keep taking the medication. If we stop them though we won't start them again. In the emergency room, what did they tell you when they offered you a stent?

Family (F): He was not offered anything.

P: I felt so bad.

Dr: The emergency room is really in action and reaction mode. When the patients are not doing well it's not the place for a conversation. But they could have offered to not put a stent in given your cancer, to make you comfortable and let you go.

Family: (strangled voice) In fact, what we are asking is if you had known would you have wanted the surgery?

P: (crying) I don't know. But if it happens again let me go. I don't want any more surgeries or resuscitation. It hurt so much when it blocked; I couldn't stay here. I am afraid it's going to happen again.

F: Did you understand? I think he means that he prefers having transfusions if he bleeds than having the stent block again.

Dr: […] So if I understand correctly, we continue the anticoagulants and we follow you closely. If you bleed or if your hemoglobin decreases we stop everything.

P: In all cases I'm really proud to be supported like this by everybody. I don't feel alone in this.

Dr: So we continue and if it bleeds we stop.

This excerpt illustrates the difficulty inherent in making end-of-life decisions despite using all four repertoires in succession: in this example, the physician first exposed the uncertainty surrounding the decision by referring to a lack of consensus between the physicians involved, then attempted to co-construct patient preferences by explicitly asking about treatment modalities such as having weekly blood tests, and affirmed patient autonomy by framing the decision as belonging to the patient. When the patient tried to uphold the authority of health care professionals and defer decision-making responsibility to clinicians, the physician emphasized the patient's autonomy even more directly by stating that it was his life and his decision. It was not until a family member intervened that a decision was finally reached. In the context of this study, strong arguments such as the uncertainty of patients’ life expectancy, their personal experiences with therapeutic options, and the fact that decisions were inherently about “their lives” made it difficult for patients to justify a decision role that was non-participative.

The discursive construction of patient participation in palliative care decisions had clinical consequences in this study. Patient participation had an impact on the legitimacy of the decisions being made. This was particularly visible during interdisciplinary meetings, where one of the questions frequently raised was: “What did the patient have to say about this decision?.” Involving patients in decision-making clearly justified the decisions being made, especially when reporting back to other members of the palliative care team. The repertoires used to construct participation in decision-making also shaped how patients died because they directly impacted the interventions that were decided on. The palliative care decisions explored throughout this study not only affected patients’ well-being, but also determined whether patients were more likely to die from blood clots or hemorrhage, whether or not they would be resuscitated, whether pneumonia would be fatal, and whether they would die at home or in an institution, to name only a few of the decisions explored.

## Discussion

In this study, we first explored how patient participation was justified through the use of interpretive repertoires during clinical interactions. We then focused on how these discursive practices conferred power over decision-making processes, thus establishing the decision roles of both palliative care providers and patients, and conferring legitimacy to decisions shaping patients’ deaths.

The first repertoire concerned the uncertainty involved in decision-making. Exposing uncertainty suggested a disinclination to assert professional authority, because it undermined the medical rationale for choosing a specific option. Exposing uncertainty was a justification for engaging patients in decision-making, to the point that when patients' participation was limited, the interactions were problematic for health care providers and decisions were difficult to defend. While SDM is deemed least controversial in the context of medically equivalent options (Elwyn, Frosch, & Rollnick, 2009; Müller-Engelmann et al., 2013), we suggest that the lack of curative options in palliative care might also increase the imperative of patient participation. Palliative care practice encompasses both a certain outcome, i.e. death, and overwhelming uncertainty about other important clinical outcomes, such as the timing of death and how it will unfold. Although participating health care providers did mention a desire for more evidence in the context of their practice, when discussing patient cases among professionals, the legitimacy of decisions was more often built on the acceptability of different options according to patients. It can be argued that this discursive practice offers a way of coping with the irreducible nature of uncertainty in medical practice towards the end of life (Han, Klein, & Arora, 2011).

Patient participation in decision-making was largely achieved through the co-construction of patient preferences. In this study, patients rarely expressed a pre-existing preference for treatment options directly. Rather, preferences were actively co-constructed through discussions of non-medical arguments, particularly by exploring patients’ previous experiences or the inconvenience of staying in the hospital for treatment. Previous work about illness narratives demonstrates the complexity of patient experiences and the need to value their cultural knowledge regarding illnesses (Groleau, 2011; Groleau, Young, & Kirmayer, 2006). Direct prompts for patients’ opinions were observed in most of the consultations during the field research. Decision co-construction and consensus were important, as justifying treatments decisions was difficult when patients withheld support for recommendations or questioned the rationale for undergoing an intervention. The need to obtain patient agreement before closing the consultation has been remarked in other clinical contexts (Koenig, 2011; Stivers, 2005). In fact, the need to take patient preferences into consideration tends to be beyond question in clinical discourse (Boivin, Green, van der Meulen, Légaré, & Nolte, 2009). That being said, our analysis represents a different way of approaching patient preferences, serving as a rhetorical purpose for giving legitimacy to decisions rather than as a pre-existing patient characteristic matched with clinical decisions.

The repertoire of affirming patient autonomy presented the patient as the rightful decision-maker. It was used as a rhetorical tool by physicians to resist patients’ attempts at deferring decisions to health care providers. When patients highlighted the lack of respect for their autonomy, it also contributed to reintroducing the co-construction of patient preferences. Positioning oneself as powerless thus represented an indirect way for patients to make health care providers revisit the notion of choice. Two important findings of this study are that health care providers never openly undermined patient choice, and that it was acceptable to frame decisions as inherently belonging to patients during the clinical encounter. In the current practice of palliative care, dying tends to be approached as an individual experience rather than as a social or religious one as it was in the past (Kellehear, 2007; McNamara, 2004). The discourse of choice has thus made its way into this palliative care context, much as it has in other clinical contexts (Mol, 2008).

Finally, the repertoire of upholding professional authority highlighted patients’ deference to health care professionals’ medical knowledge and expertise. Patients used this repertoire to explicitly defer decision-making responsibility, but also to convey respect after expressing strong opinions. It has been previously demonstrated that cancer patients use discursive practices to soften the perceived “threat” to the therapeutic relationship that their involvement in decision-making represents (Ainsworth-Vaughn, 1998). This work also supports the conclusions of previous research in primary care (Koenig, 2011; Robertson et al., 2011), insofar as the performance of patient participation remained mostly indirect, such as indirectly challenging recommendations with questions, and positioning oneself as powerless as a means of reintroducing the notion of choice. Finally, when patients exhibited a great deal of distress, professionals resolved decision-making rapidly by taking responsibility. This suggests that patient participation sometimes had to be downplayed to achieve other therapeutic goals such as reassuring patients, which has been promoted by critics of simplistic accounts of patient choice and autonomy (Ceci & Purkis, 2009; Mol, 2008; Sinding et al., 2010).

Altogether, these results show how interpretive repertoires contribute to justify patient participation in decision-making and to negotiating control over decisions that affected the ways in which patients died. It was apparent during interdisciplinary meetings that it was difficult to justify interventions that would affect the cause and timing of death when patients had not been involved in these decisions. The repertoires were combined in specific ways to confer legitimacy to the decisions, and health care providers hastened to offer excuses when clinical interactions did not exhibit patient participation in decision-making. Moreover, this study suggests that with the interpretive repertoires available, it has become difficult to defend paternalistic decision roles, except in the event of significant end-of-life distress.

This study makes a number of contributions to the field of SDM. First, it proposed a discursive conceptualization of decision-making interactions, and explicitly examined how patient participation in decision-making was justified in clinical discourse. This approach suggests that patient participation is implicitly constructed through discourse during the clinical encounter, thus complementing the SDM concepts that build on the notion of stable decision role preferences to be elicited and respected explicitly. Second, this work contributes to the scarce literature directly observing decision-making interactions in the context of palliative care. The methodology adopted was also innovative, combining descriptive analytical methods from ethnography to understand the context of the palliative care interactions observed, with methods from critical discursive psychology to unpack the discourse of patient participation. Finally, our investigation addressed the functions of discursive practices in conferring power over decision-making processes, thus shaping the roles that both palliative care providers and patients play in making decisions that affect the timing and manner of patients’ deaths.

The aim of this study was not to provide ethical guidelines for the involvement of patients in palliative care decisions. Rather, it was to expose the unforeseen and somewhat unavoidable ways in which discursive practices prompt or impede patient participation during these interactions. Discourse analysis calls for a coherence between the ethical stance that is taken by health care providers and patients about their respective decision roles, and the discursive practices they draw on during decision-making processes. Few clinicians would ever advocate for absolutely no involvement of patients in decision about their care. However, many might not be aware that the way they frame options and introduce decisions either promotes or hinders patients’ ability to justify their participation in decision-making. Considering that clinical interviewing constitutes an important dimension of teaching residents, it seems reasonable to address and integrate these issues into the training of residents.

### Rigour and Limitations

The following verification strategies were adopted during the research process in order to ensure the reliability and validity of the results: 1) methodological coherence; 2) sampling sufficiency; and 3) developing a dynamic relationship between sampling, data collection and analysis (Morse et al., 2002). First, the research question was designed specifically for a discursive study of SDM, and we expanded upon the epistemological and methodological assumptions of the research. Sampling adequacy is where this study faced most threats to validity, given the study population. Nonetheless, an effort was made to observe the work of all the health care providers on the team and to recruit a diverse sample when it came to diagnosis, illness trajectories and socioeconomic level in order to improve the validity and reliability of the results. Some patients labelled as ‘difficult’ by staff members were also recruited to avoid focusing only on highly proactive patients. Data were also collected and analyzed concurrently. In fact, data analysis encompassed several iterations, constantly checking transcripts and notes to make sure that the interpretation could be sustained across the corpus of data. The article was also sent to participating health care providers for feedback, although this could not be done with the participating patients because they were deceased by the end of the study.

Despite these efforts, this study is not without limitations. First, common limitations relating to working with a very sick and vulnerable patient population were encountered. Many patients were not recruited because they were delirious or experienced too much pain and suffering. Ascertaining the transferability of palliative care research remains a challenge in the field given the variability that exists across countries in the provision of end-of-life care (Currow, Wheeler, Glare, Kaasa, & Abernethy, 2009). The description of the sample and of the context of care aims to better situate the research findings. In addition, the participation of family members in decision-making processes was beyond the scope of this study, but more research is needed on this important topic particularly for patients with cognitive decline.

## Conclusion

In conclusion, this study identified major interpretive repertoires used to justify patient participation in palliative care decisions and examined how they were used to exert control over decision-making. These repertoires called on existing arguments and sources of legitimacy to build patient participation, such as exposing the limitations of medical evidence, and discussing treatment modalities in the context of patients’ everyday lives. Decision-making conversations had an impact on the decision roles of both palliative patients and care providers and influenced how patients ultimately died. Our work demonstrates that discursive practices deserve more attention because they shape patients’ dying trajectories in profound ways.

## Appendix A: Transcription Key for Audio-recorded Excerpts

**Table:**

Adapted from Wood and Kroger (2000, p. 193)	
[talk] [overlap]	Overlapping talk
(.)	One second pause
(2s)	Timed pause in seconds
end of line=	No interval between utterances
=start of line	
?	A rising intonation in speech delivery
!	Exclamation in speech delivery
(laugh)	Contextual information and non-speech sounds
CAPITALS	Louder than surrounding talk
*Italics*	Softer than surrounding talk
Underline	Emphasis
[…]	Talk omitted from the segment
[name omitted]	Name omitted to protect the anonymity of participants
